# Transgenic mouse lines for non-invasive ratiometric monitoring of intracellular chloride

**DOI:** 10.3389/fnmol.2013.00011

**Published:** 2013-05-21

**Authors:** Laura Batti, Marat Mukhtarov, Enrica Audero, Anton Ivanov, Rosa Chiara Paolicelli, Sandra Zurborg, Cornelius Gross, Piotr Bregestovski, Paul A. Heppenstall

**Affiliations:** ^1^Mouse Biology Unit, European Molecular Biology LaboratoryMonterotondo, Italy; ^2^Inserm UMR1106, Brain Dynamics Institute, University Aix-MarseilleMarseille, France; ^3^Laboratory of Neurobiology, Department of Physiology of Human and Animals, Institute of Fundamental Medicine and Biology, Kazan Federal UniversityKazan, Russia

**Keywords:** fluorescent biosensors, intracellular chloride, non-invasive monitoring, optogenetics, brain slices, dorsal root ganglia, macrophages

## Abstract

Chloride is the most abundant physiological anion and participates in a variety of cellular processes including trans-epithelial transport, cell volume regulation, and regulation of electrical excitability. The development of tools to monitor intracellular chloride concentration ([Cl_i_]) is therefore important for the evaluation of cellular function in normal and pathological conditions. Recently, several Cl-sensitive genetically encoded probes have been described which allow for non-invasive monitoring of [Cl_i_]. Here we describe two mouse lines expressing a CFP-YFP-based Cl probe called Cl-Sensor. First, we generated transgenic mice expressing Cl-Sensor under the control of the mouse Thy1 mini promoter. Cl-Sensor exhibited good expression from postnatal day two (P2) in neurons of the hippocampus and cortex, and its level increased strongly during development. Using simultaneous whole-cell monitoring of ionic currents and Cl-dependent fluorescence, we determined that the apparent *EC*_50_ for Cl_i_ was 46 mM, indicating that this line is appropriate for measuring neuronal [Cl_i_] in postnatal mice. We also describe a transgenic mouse reporter line for Cre-dependent conditional expression of Cl-Sensor, which was targeted to the Rosa26 locus and by incorporating a strong exogenous promoter induced robust expression upon Cre-mediated recombination. We demonstrate high levels of tissue-specific expression in two different Cre-driver lines targeting cells of the myeloid lineage and peripheral sensory neurons. Using these mice the apparent *EC*_50_ for Cl_i_ was estimated to be 61 and 54 mM in macrophages and DRG, respectively. Our data suggest that these mouse lines will be useful models for ratiometric monitoring of Cl_i_ in specific cell types *in vivo*.

## Introduction

Genetically encoded probes have become powerful tools for fluorescent analysis of the function and concentration of multiple intracellular ions and proteins (Bregestovski and Arosio, 2012; Depry et al., 2013; Perron et al., 2012). GFP derivatives with different colors have been successfully used to monitor Ca^2+^ (Miyawaki et al., 1997; Ohkura et al., 2012), pH (Kneen et al., 1998; Llopis et al., 1998; Miesenbock et al., 1998; Li and Tsien, 2012) and protein–protein interactions (Heim, 1999).

Over the last decade, several genetically encoded Cl-sensitive probes for measuring intracellular Cl concentration ([Cl]_i_) have also been developed (Kuner and Augustine, 2000; Markova et al., 2008; Arosio et al., 2010). The first generation of these probes was based on the fact that the fluorescence intensity of yellow fluorescent protein (YFP) is quenched by increasing concentrations of Cl ions (Wachter and Remington, 1999). A further improvement on this approach was the design of ratiometric Cl indicators that allowed quantitative measurements independent of the expression level of the probe. This was first achieved by designing fusion constructs of YFP coupled to cyan fluorescent protein (CFP) through a polypeptide linker. CFP fluorescent intensity is independent of Cl concentration and thus acts as a reference point for normalizing expression levels (Kuner and Augustine, 2000). This probe, called Clomeleon, has been used for measurements of [Cl]_i_ in cultured hippocampal neurons (Kuner and Augustine, 2000), in plant cells (Lorenzen et al., 2004) and in cells of the retina and brain slices (Duebel et al., 2006; Pond et al., 2006).

A number of transgenic mouse lines have been created by insertion of pH/Cl-sensitive YFP (Metzger et al., 2002) or Clomeleon (Berglund et al., 2008) DNA into the mouse genome. The most successful of these approaches relied upon random integration of a Clomeleon construct containing a Thy1 mini promoter to drive expression in sub populations of neurons (Berglund et al., 2008). These mice have allowed imaging of Cl dynamics in inhibitory circuits of different brain areas (Berglund et al., 2008; Glykys et al., 2009) and in intact hippocampus (Dzhala et al., 2012).

A disadvantage of the Clomeleon sensor is that at physiological pH it has a rather low sensitivity to Cl. The apparent *EC*_50_ of Clomeleon is more than 100 mM (Kuner and Augustine, 2000; Duebel et al., 2006) which is outside the range normally encountered in cells ([Cl]_i_: 3–60 mM) (Bregestovski et al., 2009). Moreover, attempts to generate mouse lines with inducible expression of Clomeleon in defined cell types have been hindered by low expression levels of the probe (Berglund et al., 2008).

To address these issues, we have taken advantage of a new genetically encoded indicator termed Cl-Sensor (Markova et al., 2008; Waseem et al., 2010) that contains a triple mutation in YFP which renders it more sensitive to Cl [estimated apparent *EC*_50_~ 30–50 mM (Markova et al., 2008; Waseem et al., 2010)]. We have created two mouse lines that express Cl-Sensor either under the control of the Thy1 mini promoter, or via Cre-mediated recombination from the Rosa26 locus. Here we describe the generation and characterization of these lines, and demonstrate that they allow for robust ratiometric monitoring of [Cl]_i_ across different tissues.

## Materials and methods

### Generation of transgenic mice

We generated Thy1::Cl-sensor mice because the Thy1 promoter has been demonstrated to drive robust expression in a wide variety of neurons (Caroni, 1997; Arenkiel et al., 2007). Cl-Sensor cDNA was obtained from a Cl-Sensor expression vector (Markova et al., 2008) after AfeI-HincII digestion and blunt cloned into the XhoI site of the mouse Thy1.2 expression cassette (Caroni, 1997). Linearized, vector-free insert was prepared for pronuclear injection into C57BL/6J × DBA zygotes by agarose gel purification. Two founders carrying the transgene were identified and genotyped by PCR using the following primers: Thy1 forward 5′-TCTGAGTGGCAAAGGACCTTAGG -3′ and Cl-Sensor linker reverse 5′-TCCTTGGAAGTACAAATTCTC -3′.

The cre-inducible Cl-Sensor construct was generated and assembled into the XbaI site of the Rosa26 targeting vector pROSA26 (Soriano, 1999) using classical recombinant DNA technology. ROSA26 locus was chosen because of the high efficiency of targeting by homologous recombination and because this locus gives ubiquitous expression across many tissues. The Cl-Sensor cassette consisted of the following individual sequence elements: a cytomegalovirus-immediate early (CMV-IE) promoter and a chicken beta-actin promoter (CAG); the adenovirus splice donor (SD) and splice acceptor (SA) site from plasmid pSAbgeo; an inverted wild-type loxP site (Sauer, 1987), a promoter-less neomycin resistance gene from plasmid pMC1NeopA (Thomas and Capecchi, 1987) including a Kozak consensus sequence (Kozak, 1987) followed by two successive polyadenylation sites from the bovine growth hormone gene; a mutant loxP2272 site (Siegel et al., 2001); the previously described Cl-Sensor cDNA (Markova et al., 2008) followed by the SV40 polyadenylation site, both sequences in reverse orientation relative to neomycin transcription; a wild-type loxP site and finally a mutant loxP2272 site in reverse orientation. The targeting vector was electroporated into A9 embryonic stem cells (ESC) and homologous recombinants identified by Southern blotting. Digested DNA using Bgl1 and BspHI underwent hybridization with a 3′ and 5′ probe, respectively. Using a 3′ probe two DNA fragments were visualized: a 6668 bp (wild-type fragment) and a 9329 bp mutant fragment. Using a 5′ probe two DNA fragments were visualized: a 7210 bp (wild-type) fragment and 10450 bp (mutant) fragment. A correctly identified clone was used for injection into C57BN/6 blastocysts followed by implantation of injected blastocysts into CD1 foster mothers, and backcross of male chimaeras with C57BL/6 females. All animal protocols were approved by the Italian Ministry of Health.

### Slice preparation and electrophysiological recording

Brain slices were prepared from postnatal day P3–P21 transgenic mice of both sexes. All animal protocols conformed to the French Public Health Service policy and the INSERM guidelines on the use of laboratory animals. Animals were rapidly decapitated and brains removed. Sagittal slices (300 μm) were cut using a tissue slicer (Microm International, Germany) in ice-cold oxygenated modified artificial cerebrospinal fluid (ACSF), with 0.5 mM CaCl_2_ and 7 mM MgSO_4_, in which Na^+^ was replaced by an equimolar concentration of choline. Slices were then transferred to oxygenated standard ACSF containing (in mM): 126 NaCl, 3.5 KCl, 1.2 NaH_2_PO_4_, 25 NaHCO_3_, 1.3 MgCl_2_, 2.0 CaCl_2_, and 10 D-glucose, pH 7.4, at room temperature (20–22°C) for at least 1 h before use. During recordings, slices were placed in a conventional fully submerged chamber superfused with ACSF (32–34°C). Pyramidal cells in neocortical layers and CA1 and CA3 hippocampal regions were recorded. Whole-cell patch-clamp recordings in voltage-clamp mode were performed using the EPC-9 amplifier (HEKA Elektronik, Germany). The patch pipette solution contained (mM): KCl (0–135) or KGluconate (0–135); MgCl_2_ 2; MgATP 2, HEPES/KOH 10, BAPTA 1; pH 7.3; 290 mOsm. Combination of KGluconate and KCl at a constant K^+^ concentration of 135 mM was used for Cl calibration of Cl-Sensor in Thy1::Cl-Sensor mice by five different Cl concentrations in pipette solution: 4, 10, 20, 60, and 135 mM. Pipettes were pulled from borosilicate glass capillaries (Harvard Apparatus Ltd, USA) and had resistances of 5–7 MΩ. Upon transition from cell-attached to whole-cell configuration, the holding potential was usually −80 or −70 mV.

### Cell culture and whole mount preparation

#### Dissociated DRG neurons

DRG primary cell cultures were prepared from Avil-Cre::Cl-Sensor adult mice (8–20 weeks) as previously described (Caspani et al., 2009). Briefly, mouse DRG were dissected and incubated with 1 mg/ml collagenase IV (Sigma, Italy) for 30 min at 37°C and with 0.05% trypsin (GIBCO, Italy) for 30 min at 37°C. The DRG were suspended in DMEM (GIBCO, Italy) containing 10% heat-inactivated horse serum (GIBCO, Italy), 100 U penicillin, and 100 μg/ml streptomycin (GIBCO, Italy). DRG were dissociated using 1000 and 200 μl pipet tips, and debris was removed with a 40 μm cell strainer (BD Biosciences Europe, Belgium). Cells were plated in 100 μl of medium on poly-L-lysine (100 μg/ml, Sigma, Italy) coated 35 mm ø glass bottom dish (Ibidi, Martinsried, Germany) and left to adhere for 3 h before the addition of 2 ml of medium. Experiments were conducted 24–48 h after plating of cells.

#### Whole mount DRG preparation

DRG were isolated from Avil-Cre::Cl-Sensor adult mice (8–20 weeks). DRG, together with ventral and dorsal fibers (1 cm length each side) where rapidly dissected. During the dissection tissue was wet with cold and oxygenated ACSF of the following composition (in mM): NaCl 120; NaHCO_3_ 26; NaH_2_PO_4_ 1.25; KCl 2.5; Glucose 10; MgSO_4_ 2; CaCl_2_ 2. Isolated DRG were transferred to a holding chamber containing ACSF at room temperature (20–21°C), bubbled with 95% O_2_/5% O_2_ and left to recover for at least 30 min. The whole mount DRG was then transferred to an imaging chamber and a U-shaped stainless steel rod with 12 pieces of fine nylon filaments crossing from one side to the other was used to gently hold the ganglion in place within the imaging chamber. Tissue was continuously perfused with ACSF bubbled with 95% O_2_/5% CO_2_ from a 100 ml reservoir at a flow rate of 2.5 ml/min at room temperature.

#### Peritoneal macrophage primary cell cultures

Peritoneal macrophage primary cell cultures were prepared from LysM-Cre::Cl-Sensor adult mice (8–20 weeks). Briefly, peritoneal macrophages were harvested via intraperitoneal lavage with 5 ml of DMEM (GIBCO, Italy). Cells where plated in 200–400 μl of DMEM medium on a petri dish and left to adhere for 2–3 h. After one wash with medium to remove the non-adherent cells, cells were incubated on DMEM supplemented with 10% heat-inactivated horse serum and 100 U penicillin, and 100 μg/ml streptomycin (GIBCO, Italy) at 37°C 24–48 h before proceeding with the experiments.

For basal intracellular chloride measurements DRGs and macrophages cultures were kept in a HEPES buffer solution of the following composition (in mM): NaCl 140; NaOH 4.55; HEPES 10; KCl 4; Glucose 5; MgCl_2_ 1; CaCl_2_ 2. pH 7.4. For basal measurement on whole mount DRG, preparations were transferred to the imaging chamber described above and continuously perfused with oxygenated ACSF of the following composition (in mM): NaCl 120; NaHCO_3_ 26; NaH_2_PO_4_ 1.25; KCl 2.5; Glucose 10; MgSO_4_ 2; CaCl_2_ 2.

### Calibration of Cl-sensor in Rosa26::Cl-sensor mice

To calibrate the intracellular Cl dependence of the Cl-Sensor we used dissociated DRGs culture and peritoneal macrophages culture. Experiments were assessed in 1–2 days old cultures. All imaging experiments were conducted at 37°C in a humified chamber perfused with 5%CO_2_. Different concentrations of Cl in the extracellular solution were created by mixing two “high potassium” solutions, containing in mM: (i) 164.8 KCl, 10 d-glucose, 20 HEPES, pH 7.3 and (ii) 164.8 K-gluconate, 10 d-glucose, 20 HEPES, pH 7.3. To increase the permeability of the cell membrane to Cl ions, 40 or 80 μM β-escin (Sigma, Italy) was added to neuronal or macrophages cultures, respectively. β-escin was dissolved in water and prepared freshly for each experiment. This suspension was stable for about 2 h. Cells were incubated with β-escin for a maximum of 2 min and then intensely washed with HEPES buffer solution of the following composition (in mM): NaCl 140; NaOH 4.55; HEPES 10; KCl 4; Glucose 5; MgCl_2_ 1; CaCl_2_ 2. pH 7.4. The coverslip with cultured cells was then placed into the recording chamber and incubated with high potassium extracellular solution containing a given Cl concentration (from 0 to 150 mM Cl at pH 7.3). After a stabilization of the fluorescence, imaging experiments were carried out at the following Cl concentration in the extracellular solution: 0, 10, 20, 30, 50, 100, and 150 mM. The fluorescence responses of Cl-Sensor corresponding to specified Cl concentrations inside the cell were registered. Fluorescence data was fit with a Hill equation, which describes a sigmoidal curve.

$$Y=A+\frac{B\times x^{H}}{EC50^{H}+x^{H}}$$

Where *y* is the fluorescence (440/514 nm) value and *x* the [Cl], *A* is the lowest 440/514 nm value, *B* the highest 440/514 nm value, *EC*_50_ is the midpoint of the curve and *H* is the Hill coefficient. Values of Cl concentrations were then calculated according to the inverse function:

$$\left[Cl\right]={\left(\frac{EC50^{H}}{\frac{B}{y-A}-1}\right)}^{1/H}$$

For calibration of macrophages in culture the values were the following: *A* = 0.91, *B* = 1.97, *EC*_50_ = 60.79 mM and *H* = 4.46. For calibration of DRG cultures the variables were the following: *A* = 0.90, *B* = 2.59, *EC*_50_ = 54.46 mM and *H* = 3.12.

### Real-time fluorescence imaging and image analysis in cells from Rosa26::Cl-sensor mice

Dissociated DRG and macrophages were plated onto 35 mm ø glass bottom dishes (Ibidi, Martinsried, Germany) and maintained using a microscope cage incubator, which allowed for a constant temperature (37°C), humidity and CO_2_ (5%). Whole mount DRG and hippocampal slice were placed in the imaging chamber and continuously perfused with oxygenated ACSF at a flow rate of 2.5 ml/min at room temperature. Time Lapse video-microscopy was carried out using a Spinning Disk confocal Ultraview Vox (Perkin Elmer), interfaced with Volocity 6.0 software (Cellular imaging, Perkin Elmer). Diode solid state lasers, operating at 440 and 514 nm were used as excitation sources for the CFP and YFP, power was set at the same percentage for both lasers. Band pass emission filter-cubes of 485 (W60) and 587 (W125), were used for the acquisition.

Images were acquired using a Hamamatsu EMCCD camera; exposure time and camera sensitivity were set equal for both channels. The recording protocol was designed with an initial equilibration time of the 440/514 nm ratio, and afterwards images were acquired every minute. The duration of excitation for the two lasers was 606 ms.

Images analysis was carried out using ImageJ. Ratios of images acquired upon excitation at 440 nm and those obtained upon excitation at 514 nm (440/514) were calculated by dividing the two thresholded images. Mean gray intensity value for each cell was then calculated manually. Results are presented as mean ± SEM. The data were analyzed by Student's *t*-test. Pearson's correlation analysis was used to correlate cell size and intracellular chloride concentration. A *p*-value < 0.05 was accepted as significant.

### Real-time fluorescence imaging of neurons in slices from Thy1::Cl-sensor mice

Fluorescence images were acquired using a customized digital imaging microscope. Excitation of the CFP and YFP in Cl-Sensor expressing cells in slices from Thy1::Cl-Sensor mice at wavelengths of 440 and 480 nm was achieved using a 1-nm-bandwidth polychromatic light selector equipped with a Polychrome V (150 W xenon lamp, Till Photonics, Germany). Light intensity was attenuated using neutral density filters. A dichroic mirror (495 nm; Omega Optics, USA) was used to deflect light onto the samples. Fluorescence was visualized using an upright microscope (Axioskop) equipped with a 60× water-immersion objective (n.a. 0.9; LumPlanFL, Olympus, USA). Fluorescent emitted light passed to a 16-bit electron multiplying charge-coupled device digital camera system equipped with an image intensifier (Andor iXon EM+; Andor Technology PLC, Northern Ireland). Images were acquired on a computer via a DMA serial transfer. All peripheral hardware control, image acquisition and image processing were achieved using customized software iQ (Andor Technology PLC, Northern Ireland). The average fluorescence intensity of each region of interest (ROI) was measured. Mean background fluorescence (measured from a non-fluorescent area) was subtracted and the ratio (*R*) intensities *F*_440_/*F*_480_ (mentioned above) were determined. Sampling interval was usually 10 or 5 s in some cases and duration of excitation was 10–20 ms.

## Results

### Generation of chloride sensor transgenic mice

We generated two transgenic mouse lines for Cl-Sensor, using either the mouse Thy1 mini promoter or a Cre-dependent inducible approach.

### Thy1 mice expressing Cl-sensor

**Figure 1:**
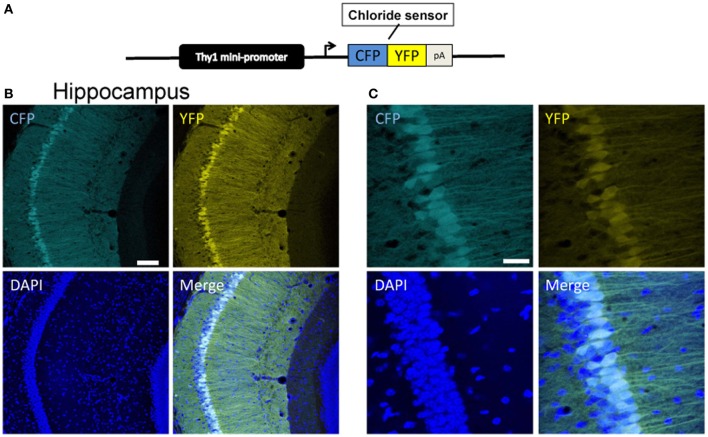
**Expression of Cl-Sensor in *Thy1*::Cl-sensor transgenic mice. (A)** The *Thy1*::Cl-Sensor construct was constructed by fusing a fragment containing minimal regulatory elements and the promoter of the *Thy1* gene upstream to the coding sequence of the Cl-Sensor followed by a SV40 polyadenylation signal. (**B** and **C**) Representative z-stack projection images of the CA1 region of the hippocampus in a *Thy1*::Cl-Sensor transgenic mouse showing CFP (upper left), YFP (upper right), and DAPI (lower left), and merged (lower right) fluorescence at **(B)** 20× magnification, scale bar corresponds to 30 μm and **(C)** 40× magnification, scale bar corresponds to 100 μm. Cl-Sensor expression was found in a mosaic pattern in most, but not all CA1 pyramidal neurons.

**Figure 2:**
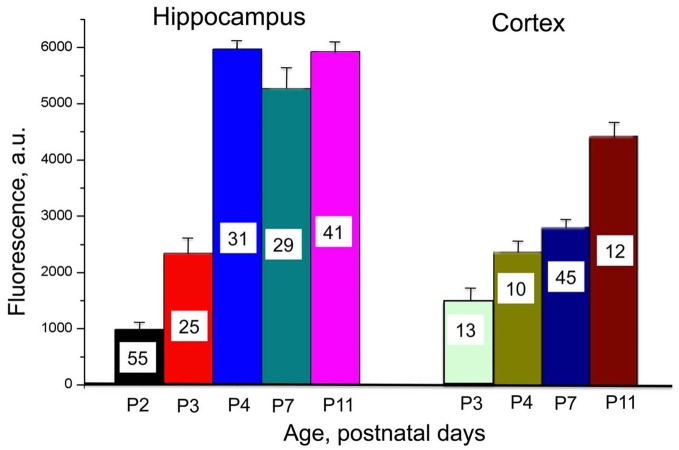
**Developmental profile of Cl-Sensor expression in hippocampus and cortex in Thy1::Cl-Sensor transgenic mice.** Mean values of fluorescence induced by excitation of neurons from brain slices in hippocampus (left) and cortex (right) at different ages (shown below columns). An excitation wavelength of 440 nm for a duration of 20 ms was used with a ×60 objective. Bars are mean ± SEM values. Number of analyzed cells for each age is shown in columns.

In the first approach, we used an expression cassette containing a 6.5 kb genomic DNA fragment of the *Thy1* gene extending from the promoter to exon 4, but where exon 3 and its flanking introns were replaced by a XhoI linker (Caroni, 1997). This vector has been shown to drive strong constitutive transgene expression in neurons of postnatal (P6–12) and adult mice (Aigner et al., 1995). The Cl-Sensor cDNA was cloned at the level of the XhoI site and the purified insert *Thy1*::Cl-Sensor was injected into C57BL/6J × DBA pronuclei (Figure 1). Two transgenic founders were identified and analyzed for Cl-Sensor expression. Expression was observed from P2 and increased strongly with development. At P2–P22 Cl-Sensor fluorescence was observed in hippocampus, particularly in CA1 and CA3, and neocortex (Figures 1A,B, 2, 4A,B, 5A).

We performed a comparative analysis of Cl-Sensor expression in hippocampus and cortex by monitoring fluorescence in brain slices from animals of different ages (from P2–P3 to P11) during excitation of the Cl-independent CFP component (with 440 nm wavelength) and recording in identical conditions. As illustrate in Figure 2, robust increase in the fluorescence was observed in hippocampus, which reached a maximal level at P4. In contrast, in the cortex the Cl-Sensor expression developed slower and even at P11 it expression was lower than in hippocampus (Figure 2).

### Cre-inducible Cl-sensor mice

**Figure 3:**
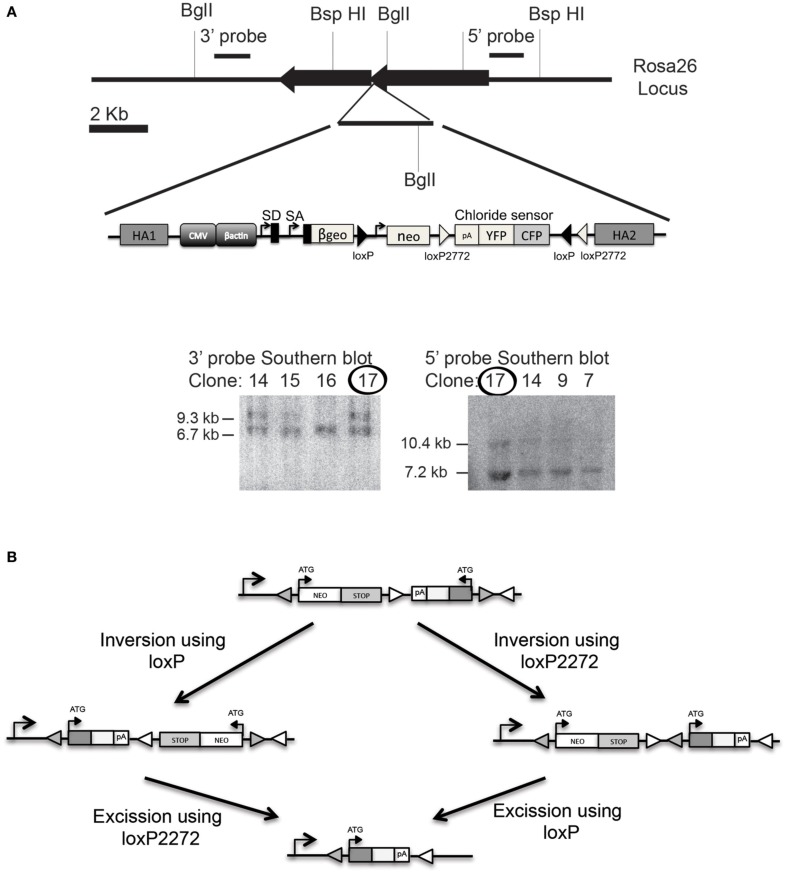
**Generation of Cl-Sensor transgenic mouse. (A)** The Cl-Sensor cassettes were assembled and inserted into the XbaI site of the ROSA26 targeting vector. Southern blot was used to confirm proper homologous recombination on ES cell selected clones. Clone number 17 was selected for blastocyst injection. **(B)** Schematic representation of the Rosa26 locus after targeted introduction of the neomycin-Cl-Sensor cassette. The Cl-Sensor cassette, including an SV40 polyadenylation site, is inserted in an antisense orientation. Two alternative recombination intermediates are generated by Cre-mediated inversion at the wild-type loxP sites (filled triangles), and mutant loxP2272 sites (open triangles).

To generate an inducible Cl-Sensor line a Cre-inducible Cl-Sensor-cassette was targeted to the Rosa26 locus by homologous recombination in ESC (Figure 3). The targeting construct was designed to give strong expression of Cl-Sensor in the absence of leakiness. To this end we included a strong CAG promoter upstream of the coding sequence and inserted the Cl-Sensor in an inverted orientation. As shown in Figure 3B the specific position of the two loxP sites allows a Cre-dependent inversion of the intervening sequences at either the loxP or loxP2272 sites, followed by irreversible excision of the neomycin stop cassette along with its loxP or loxP2272 site (Luche et al., 2007). Southern Blot confirmed appropriate homologous recombination on ES cell targeted clones and positive clones were selected for blastocyst injection.

### Calibration of Cl-sensor in brain slices from Thy1 mice

**Figure 4:**
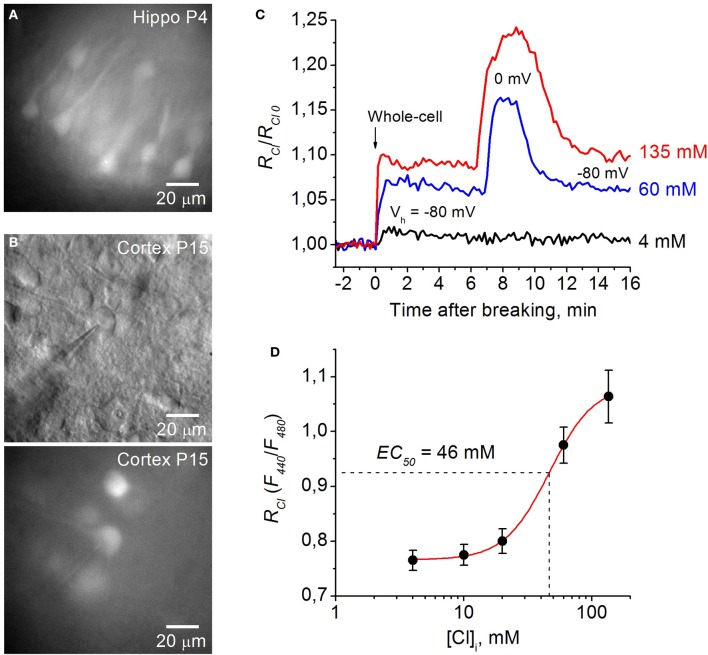
**Calibration of Cl-Sensor in brain slices of Thy1 mice. (A)** Image of cells expressing Cl-Sensor in a hippocampal slice from P4 aged Thy1::Cl-Sensor mouse, excitation 480 nm. **(B)** Micrographs of cells expressing Cl-Sensor in cortical slice from Thy1 mouse at age P15; top, light illumination; Note the shadow of the recording pipette; bottom, excitation 480 nm. **(C)** Relative changes in *R*_*Cl*_ (*F*_440_/*F*_480_) from simultaneous whole-cell recordings with different concentration of Cl in the recording pipette: 4 mM (*black trace*), 60 mM (*blue trace*), and 135 mM (*red trace*). *R*_*Cl*0_ corresponds to [Cl]_i_ in cell attached mode. Time = 0 corresponds to the moment of membrane rupture into whole-cell mode. Note the further increase in *R*_*Cl*_ at depolarization from 𢈒80 to 0 mV in the cells recorded with 60 mM (*blue trace*) and 135 mM (*red trace*) Cl in the pipette. **(D)** Calibration curve for Cl-Sensor expressed in neurons of brain slices from Thy1 mice obtained by recording at five different Cl concentrations: 4, 10, 20, 60, and 135 mM. *EC*_50_ = 46.4 ± 2.2 mM (mean ± S.E.M.). Data from 5 to 7 cells for each Cl concentration are presented.

Expression of Cl-Sensor was observed from P2 and its intensity increased with development (Figure 2). To evaluate the sensitivity of Cl-Sensor we used simultaneous monitoring of whole-cell currents and fluorescent signals in neurons from brain slices of transgenic mice (Figure 4B). Whole-cell recordings were performed with five different Cl concentrations in the pipette solution ([Cl]_p_): 4, 10, 20, 60, and 135 mM. For ratiometric estimation of [Cl]_i_, the ratio *R*_*Cl*_ = *F*_440_/*F*_480_ was used.

Figure 4C illustrates relative changes in [Cl]_i_ upon transition from cell attached to whole cell configuration from three cells in brain slices from P15 mouse using pipettes with solutions containing 4, 60, and 135 mM Cl. *R*_*Cl*0_ corresponds to [Cl]_i_ in cell attached mode, i.e., to the native concentration of Cl in cytoplasm of the recorded cell. Breaking the membrane to obtain whole-cell configuration with the pipette containing 135 mM Cl (holding potential –80 mV) resulted in an increase of *R*_*Cl*_/*R*_*Cl*0_, corresponding to elevation of [Cl]_i_. Interestingly, further increase in *R*_*Cl*_ was observed at depolarization of the cell to 0 mV (Figure 4C). This suggest that [Cl]_i_ in the cell did not reach the [Cl]_p_ value at *V*_*h*_ = −80 mV. Similar but slightly lower changes in *R*_*Cl*_ were observed in the cell recorded with the pipette containing 60 mM Cl. Rupture of the membrane with the pipette containing 4 mM Cl produced a small increase in *R*_*Cl*_/*R*_*Cl*0_, indicating that the basal value of [Cl]_i_ in the cytoplasm of the neuron was lower than 4 mM. After reaching a maximal value, usually a decrease in the ratio was observed (Figure 4C), presumably due to pumping out of Cl by transporters (Pellegrino et al., 2011). Maximal values of *R*_*Cl*_ were used for obtaining the calibration curve. For high [Cl]_p_ = 60 mM and 135 mM the *R*_*Cl*_ values at *V*_*h*_ = 0 mV were used while for lower [Cl]_p_ = 4, 10, and 20 mM the holding potential always was kept at −80 or −70 mV (Figure 4C).

In slices from Thy1::Cl-Sensor mice the calibration curve obtained from neurons recorded with pipettes containing five different [Cl]_p_ (Figure 4D) was best fit with a Logistic Dose-Response Sigmoidal curve using the OriginPro 8.5 program with the formula:

$$R_{Cl}=A2+\frac{A\text{1}-A\text{2}}{\text{1}+{\left(\frac{{\left[Cl\right]}_{i}}{K_{d}}\right)}^{p}},$$

where *R*_*Cl*_ is the fluorescence ratio for Cl (F_440_/F_480_), *K*_*d*_ is the dissociation constant for Cl binding, A1 and A2 are the minimum and maximum asymptotic values of *R*_*Cl*_, respectively, and *p* is the power value.

By rearranging this formula we obtained the equation for [Cl]_i_:

$${\left[Cl\right]}_{i}=K_{d}\;\cdot \;{\left(\frac{A\text{1}-A2}{R_{Cl}-A\text{2}}-\text{1}\right)}^{\frac{\text{1}}{p}}$$

For Thy1::Cl-Sensor the values of constants obtained from fitting the curve were the following: *K*_*d*_ = 46.4 mM, *A*1 = 0.76, *A*2 = 1.08 and *p* = 2.48 (Figure 4D).

Using this approach we determined that within a physiological range of Cl concentrations (0–135 mM) the mean apparent *EC*_50_ (concentration of Cl producing a 50% change in the fluorescence ratio, *EC*_50_) of Thy1::Cl-Sensor was 46.4 ± 2.2 mM (Figure 4D, Table 1). While the sensitivity of Cl-Sensor to Cl in Thy1::Cl-Sensor mice was similar to that reported previously (Markova et al., 2008; Waseem et al., 2010), the dynamic range of *R*_*Cl*_ changes was about 2–3 fold smaller than those obtained in CHO cells and cultured spinal neurons. The basis for these differences requires future analysis.

**Table 1:** **Table summarizing the *EC*_50_ and Hill coefficient calculated on different tissue or cell culture isolated from the mouse lines described in the manuscript**.

	**Mouse line**	***EC*_50_ (mM)**	**Hill coefficient**
Brain slice	Thy1::Cl-Sensor	46.4 ± 2.2	2.48
DRG culture	Avil-Cre::Cl-Sensor	54.46 ± 6.3	3.12
Macrophages culture	LysM-Cre::Cl-Sensor	60.79 ± 3.96	4.46

### Examples of monitoring [Cl]_i_ in slices expressing Cl-sensor

**Figure 5:**
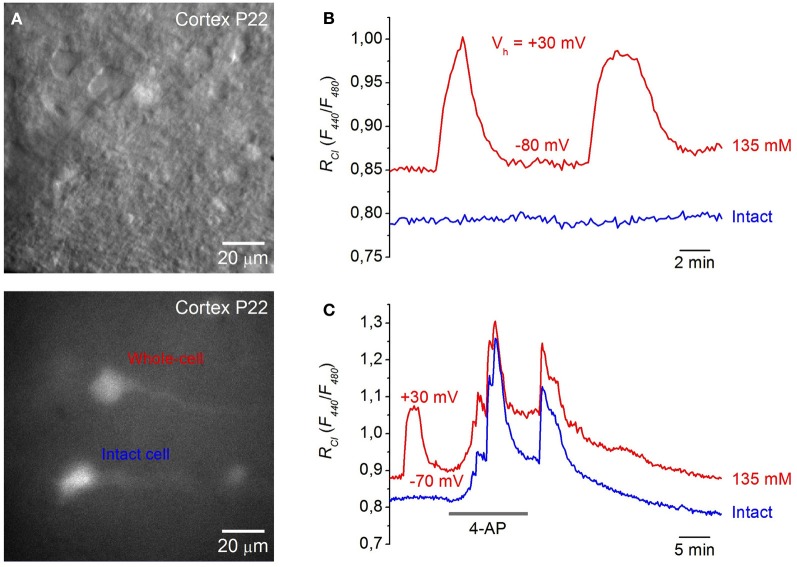
**Monitoring [Cl]_i_ in neurons from cortical brain slices. (A)** Images of cells in a cortical slice from a P22 mouse expressing Cl-Sensor in visible light (top) and excitation 480 nm (bottom). **(B)** Example of changes in *R*_*Cl*_ (*F*_440_/*F*_480_) after membrane depolarization from a holding potential of −80 to +30 mV in the neuron from a cortical slice (P15) recorded with pipette solution containing 135 mM Cl (*red trace—*whole-cell, *blue trace—*intact cell). Note that depolarization of the cell recorded with high Cl caused an additional increase in the *R*_*Cl*_. **(C)** Monitoring of epileptic-like seizures in neurons of brain slice from cortex (P22). Traces of *R*_*Cl*_ changes from two neurons are illustrated upon application of 100 μM 4-AP. The neuron corresponding to the *red trace* was patched with a pipette containing 135 mM Cl, while the *blue trace* corresponds to the record from intact neurons. Note that 4-AP application caused a stronger increase in *R*_*Cl*_ than depolarization from 𢈒70 to +30 mV.

We next monitored changes of [Cl]_i_ in neurons from brain slices of Thy1:Cl-Sensor mice under different experimental conditions. Similar to previous observations using transient transfection of Cl-Sensor into neurons (Markova et al., 2008; Mukhtarov et al., 2008), depolarization induced by bath application of 20–40 mM KCl or inhibition of voltage gated K^+^ channels caused reversible changes of [Cl]_i_ (data not shown). Moreover, as observed in CHO cells expressing Cl-Sensor and the human glycine receptor (Markova et al., 2008), application of positive potentials to neurons induced elevation of [Cl]_i_. Thus, as illustrated in Figure 5B, changes in membrane potential from −80 to +30 mV using pipettes containing 135 mM Cl induced remarkable and reversible [Cl]_i_ increase. This indicates that depolarization changes the Cl equilibrium and is able to do so even in cells containing very high Cl concentration.

It had been well documented that 4-aminopyridine (4-AP), a blocker of K^+^ channels, induces epileptoform activity associated with ictal discharges resulting from synchronous GABA-mediated depolarizing potentials. This is accompanied by transient increases of extracellular K^+^ (Avoli et al., 1996; Barbarosie et al., 2002) and elevation of intracellular Cl (Dzhala and Staley, 2003; Khalilov et al., 2003; Glykys et al., 2009). To visualize kinetics and amplitude of Cl changes in this epileptogenic model, we monitored changes of [Cl]_i_ in neocortical slices from mice expressing Cl-Sensor during epileptoform activity induced by 4-AP.

Figure 5C shows a recording from two neurons. One was patch-clamped with a pipette containing 135 mM Cl while the other was left intact. Application of 100 μM 4-AP caused a robust and reversible elevation of Cl in recorded neurons. Its amplitude was even higher than those induced by membrane depolarization to +30 mV (Figure 5C, *red trace*). Importantly, the time course and amplitude of [Cl]_i_ transients were similar in both neurons.

These initial observations indicate that transgenic mice expressing Cl-Sensor from the Thy1 promoter represent a good tool for monitoring of [Cl]_i_ transients in different experimental models.

### Chloride measurements on dissociated DRG neurons from Avil-Cre::Cl-sensor mice

**Figure 6:**
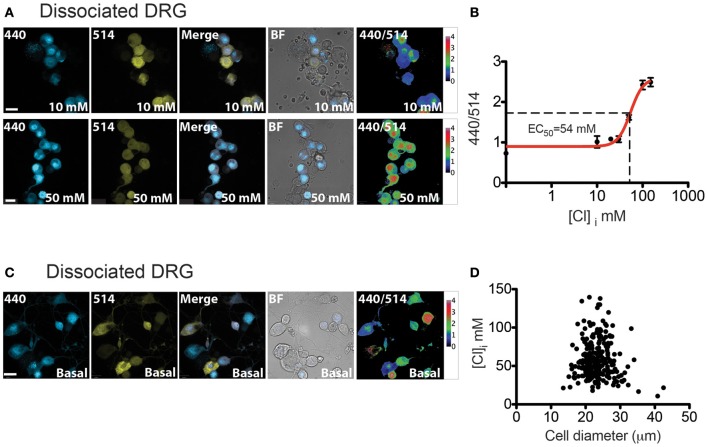
**[Cl]_i_ calibration and measurement in DRG cultures. (A)** Image examples of permeabilized DRG cultures from Avil-Cre::Cl-Sensor at 10 mM (top) or 50 mM (bottom) chloride concentration. From left to right: image captured with the 440 nm laser (blue), 514 nm laser (yellow), merged, bright field images and 440/514 images. Scale bars correspond to 20 μm **(B)** [Cl]_i_ Calibration curve for Cl-Sensor on DRG cultures from Avil-Cre::Cl-Sensor. 440/514 values were calculated at 0, 10, 20, 30, 50, 100, and 150 mM [Cl]_i_ (*n* = 55). **(C)** Examples of [Cl]_i_ measurement in DRG culture under basal conditions. From left to right: image captured with the 440 nm laser (blue), 514 nm laser (yellow), merged, bright field images and 440/514 images. Scale bar corresponds to 20 μm (**D**) The relationship between cell size and [Cl]_i_ in DRG neurons *n* = 260.

Using the inducible Rosa26 mouse line, we investigated the expression and function of Cl-Sensor in two separate cell types; peripheral sensory neurons, and cells of the myeloid lineage. To investigate the expression and function of Cl-Sensor in sensory neurons, Cl-Sensor transgenic mice were crossed with an Advillin-Cre driver line, which specifically targets sensory neurons in dorsal root ganglia and trigeminal ganglia (Zurborg et al., 2011). DRG cultures were prepared from adult Avil-Cre::Cl-Sensor mice and used for calibration of the Cl-Sensor probe (Figure 6).

In order to increase the permeability of the cell membrane to Cl ions for calibration experiments, the natural triterpenoid saponin, β-escin was applied to cells. This compound has been shown to be effective for *in situ* calibration of Cl-Sensor and to give more reliable results than other methods (Waseem et al., 2010). After treatment with β-escin (40 μM), cells were placed in isosmotic extracellular solutions containing different concentrations of Cl. Extracellular solutions were prepared by substituting equimolar concentrations of K-gluconate with KCl. A Cl-free solution containing 164.8 K-gluconate was used to determine the minimum excitation ratio of Cl-Sensor and a solution containing 164.8 KCl gave the maximum excitation ratio.

Ratiometric measurements of emission fluorescence at 440 and 514 nm excitation wavelengths (440/514) were used to calibrate the probe (Figure 6A). The estimated intracellular Cl concentration was plotted against the 440/514 ratios and the data was fitted with a Hill equation (Figure 6B). The calculated *EC*_50_ was 54.46 ± 6.3 mM (Table 1). This value resembles results described by Waseem et al., where the *EC*_50_ was 48.9 ± 6.3 mM, calculated on cultured spinal neurons transfected with the Cl-Sensor probe (Waseem et al., 2010).

We next examined basal intracellular Cl levels in DRG neurons. As illustrated in Figure 6C, calculation of the 440/514 ratio indicates that there is a broad range of estimated [Cl]_i_ amongst DRG neurons; mean [Cl]_i_ in DRG neurons is 58.16 ± 1.5 mM with a 95% of confidence interval from 55.20 to 61.11 mM, *n* = 260. DRG neurons can be broadly classified on the basis of their size into three functionally distinct populations of “small,” “medium,” and “large” neurons, which approximately correspond to C, Aδ and Aα-Aβ fibers (Study and Kral, 1996). As shown in Figure 6D, fluorescence ratio was not correlated to cell size suggesting that differences in [Cl]_i_ are not significantly different between these populations (Pearson *R* = −0.076, *p* = 0.22, *n* = 260).

### Chloride measurements on *ex vivo* whole DRG preparations from Avil-Cre::Cl-sensor

**Figure 7:**
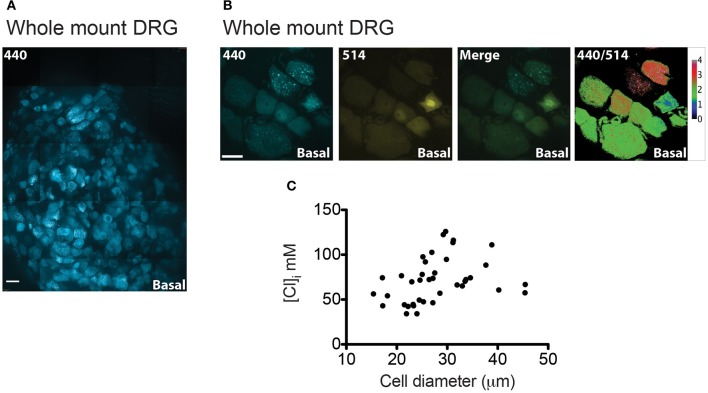
**[Cl]_i_ measurement on whole-mount DRG from Avil-Cre::Cl-Sensor mice. (A)** An xy-plane projection of a deconvolved 75 μm stack of an isolated whole-mount DRG. Tissue was excited with a 440 nm laser and CFP (blue) was detected. Scale bar corresponds to 200 μm. **(B)** Example of a DRG image under basal conditions used for [Cl]_i_ measurement. From left to right: image captured with the 440 nm laser (blue), 514 nm laser (yellow), and 440/514 images. Scale bars correspond to 20 μm. **(C)** The relationship between cell size and [Cl]_i_ in DRG neurons from a whole mount preparation *n* = 39.

The analysis of Cl-Sensor in sensory neurons was extended by utilizing an *ex vivo* whole mount preparation of DRG (Figure 7). We reasoned that this preparation may give a more accurate representation of physiological Cl levels in sensory neurons than dissociated culture. For example, three dimensional architecture, cellular connectivity and density of the ganglion would be expected to be preserved in a whole mount preparation. Furthermore, tissue is not subjected to a lengthy dissociation protocol which may modify ion homeostasis. Using this preparation, tissue was viable in oxygenated ACSF for at least 5 h.

Figure 7A shows a stitched image of a whole-mount DRG and illustrates that Cl-Sensor is robustly expressed in the majority of the DRG neurons. Analysis of [Cl]_i_ in these neurons again demonstrated a heterogeneous distribution of 440/514 ratios with a mean [Cl]_i_ of 71.58 mM and a 95% confidence interval from 63.21 to 78.24 mM, *n* = 39 (Figures 7B,C). Of note these values were significantly higher than those observed in cultured DRG neurons (*p* < 0.001) and may reflect the increased physiological relevance of this preparation compared to dissociated neurons.

### Chloride measurements on macrophages from LysM-Cre::Cl-sensor mice

**Figure 8:**
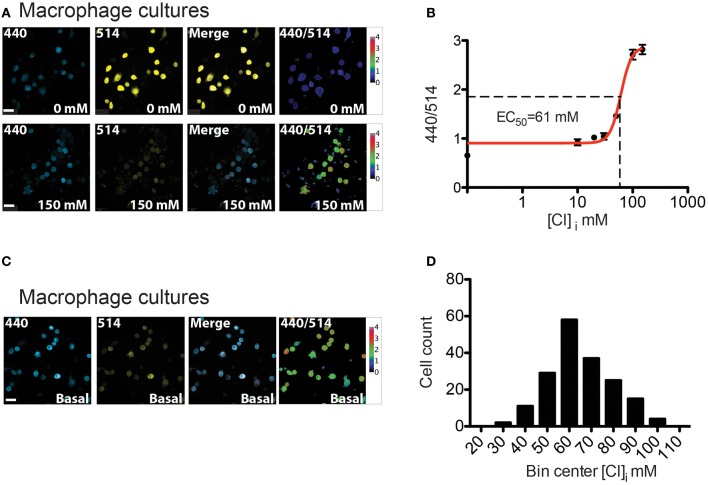
**[Cl]_i_ calibration and measurement in peritoneal macrophages from LysM-Cre::Cl-Sensor mice. (A)** Representative images of permeabilized peritoneal macrophages cultures which were subjected to β-escin treatment followed by the addition of 0 mM (top) or 150 mM (bottom) extracellular Cl^−^ solutions. From left to right: image captured with the 440 nm laser (blue), 514 nm laser (yellow), merged and 440/514 images. Scale bars correspond to 20 μm. **(B)** [Cl]_i_ calibration curve on peritoneal macrophages cultures. 440/514 values were calculated at 0, 10, 20, 30, 50, 100, and 150 mM [Cl]_i_ (*n* = 225). **(C)** Images of basal [Cl]_i_ in macrophages. **(D)** Histogram of [Cl]_i_ distribution in macrophages (*n* = 181). The distribution of [Cl]_i_ was fit with a Gaussian curve to give a mean of 65.48 ± 1.07 mM. Scale bars correspond to 20 μm.

Myeloid lineage-specific expression of Cl-Sensor was obtained by crossing Cl-Sensor transgenic mice with LysM-cre, a Cre-driver line targeting cells of the myeloid lineage (Clausen et al., 1999). We firstly assessed the expression and function of the transgene in peritoneal macrophages isolated from adult LysM-Cre::Cl-Sensor mice. Robust expression of the probe was observed in macrophages (Figure 8) that was evident in the majority of cells examined. Calibration of the probe was performed in the same way as in DRG neurons by treating cells with β-escin (80 μM) and monitoring fluorescence at different Cl concentrations (Figure 8A). In agreement with results in DRG neurons, data was fit with a Hill equation to give an *EC*_50_ of 60.79 ± 3.96 mM (Figure 8B and Table 1). While being slightly higher (but not significantly: 95% confidence of interval in DRG is 41.09–67.83, in macrophages is 53.0–68.5) than in previous calibrations this mean *EC*_50_ value suggests that although a different Cre driver line was used, the ratiometric measurement can be applied with a similar output to both models. Finally, Cl-Sensor was utilized to measure basal [Cl]_i_ in isolated macrophages. The mean concentration calculated from 181 macrophages was 65.48 with a 95% of confidence interval from 63.21 to 67.60 mM (Figures 8C,D).

## Discussion

To obtain new tools for non-invasive monitoring of intracellular Cl concentration under normal and pathological conditions, two transgenic mouse lines were generated expressing Cl-sensitive probes either in neurons, or targeted to the Rosa26 locus for inducible expression.

### Thy1::Cl-sensor transgenic mice

In the first approach, Cl-Sensor (Markova et al., 2008) was expressed under the control of the neuron-specific Thy1 promoter (Caroni, 1997; Feng et al., 2000; Berglund et al., 2008). Because of a triple mutation in YFP, this sensor displayed an enhanced sensitivity to Cl that was within the physiological range of intracellular Cl concentrations encountered in different cell types (Markova et al., 2008; Waseem et al., 2010). By simultaneously monitoring fluorescence and ionic currents in whole-cell patch clamp recordings with different concentrations of Cl in the recording pipette, we calibrated the sensor and estimated the apparent *EC*_50_ for Cl to be 46 mM (at pH 7.4). This value is close to previously reported calibration data for Cl-Sensor in cell lines and neurons (Markova et al., 2008; Waseem et al., 2010).

An early study describing the Thy1 cassette in transgenic mice reported that this promoter induces expression in neurons from around P6–P10 (Caroni, 1997). We observed fluorescence in hippocampal and neocortical neurons from P2 and its level increased substantially with development. Cl transients were observed in hippocampus (particularly in CA1 and CA3) and neocortex. Similar to previous observations (Metzger et al., 2002; Markova et al., 2008; Mukhtarov et al., 2008), depolarization induced by bath application of KCl or via the recording pipette brought about a strong elevation in intracellular Cl in neurons or brain slices.

To explore this further, the K^+^ channel blocker, 4-AP, was applied to slices. This compound is widely used as a tool for increasing neuronal network excitatory activity, and has been shown to generate interictal-like events in human neocortical tissue via depolarization and an excitatory action at GABA receptors (Avoli et al., 1994). We used this blocker for inducing seizure-like activity and monitored Cl transients under these conditions. 4-AP caused a robust elevation in [Cl]_i_, indicating that depolarization induces an increase in Cl driving force that promotes Cl influx, presumably through Cl-selective channels or via changes in the activity of Cl transporters.

Two interesting features concerning Cl transients were observed when monitoring neurons in brain slices from *Thy1*::Cl-Sensor mice. Firstly, depolarization was able to induce elevation of [Cl]_i_: changing the *V*_*h*_ to 0 mV and more positive potentials caused a strong increase in [Cl]_i_. This effect was not observed when performing similar experiments in HEK or CHO cells expressing Cl-Sensor (Markova et al., 2008), Biosensor GlyR (Mukhtarov et al., 2008) or ClopHensor (Mukhtarov et al., 2013). Secondly, in cells recorded with 135 mM Cl at a holding potential of +30 mV, the *R*_*Cl*_ value was lower than that following 4-AP application (Figures 5B,C). These observation suggests that in brain slices [Cl]_i_ does not reach [Cl]_p_ values even at *V*_*h*_ ≥ E_*Cl*_. The activity of neuronal Cl transporters (Pellegrino et al., 2011; Friedel et al., 2013) or some other reasons may be responsible for these effects. More detailed calibration analysis of *Thy1*::Cl-Sensor function in neurons in brain slices may eventually clarify these phenomena.

### Rosa26::Cl-sensor transgenic mice

In a second approach, transgenic mice with Cl-Sensor knocked into the Rosa26 locus were generated. We used an inducible strategy where the Cl-Sensor transgene was integrated into the Rosa26 locus in a reverse orientation. Due to the arrangement of loxP and mutant loxP2272 sites, Cre-mediated recombination induces an inversion of the sensor that can be exploited for tissue specific expression (Luche et al., 2007). To validate this strategy we crossed mice with a sensory neuron specific Cre-driver line, Advillin-Cre (Zurborg et al., 2011), and a myeloid lineage driver LysM-Cre (Clausen et al., 1999). In both approaches, robust expression of Cl-Sensor in DRG neurons and macrophages was observed. Mice were analyzed further to determine basal [Cl]_i_ in these different cell types.

Early reports on intracellular Cl concentration in peripheral neurons were based mainly on measurements of the reversal potential of GABA_A_ receptor mediated Cl currents. These studies demonstrated that in primary afferent neurons, GABA_A_ receptor activation is associated with membrane depolarization, indicating that intracellular Cl concentration is relatively high. (Deschenes et al., 1976; Alvarez-Leefmans et al., 1988). Indeed, [Cl]_i_ in neurons of mammalian DRG has been estimated to be in the range 30–50 mM using both electrophysiological (Alvarez-Leefmans et al., 1988; Kenyon, 2000; Kaneko et al., 2004) and fluorescent imaging methodologies (Rocha-Gonzalez et al., 2008). These values are close to those to obtain in our experiments on dissociated DRG neurons (approximately 58 mM).

The elevated [Cl]_i_ in peripheral sensory neurons appears to be due to the high expression of the Na^+^ −K^+^ −Cl^−^ co-transporter (NKCC1) in these cells. In NKCC1 knockout mice, absence of the co-transporter reverses GABA-mediated currents such that strong depolarizing responses elicited by GABA in control mice become hyperpolarizing in NKCC1 null mutants (Sung et al., 2000). Using gramicidin-perforated patch-clamp recording, it has been shown that DRG neurons from wild-type animals have a [Cl]_i_ of approximately 47 mM, while in neurons from NKCC1 knock-out mice this value is much lower at around 25 mM (Sung et al., 2000).

A “whole mount” DRG preparation was used to determine whether [Cl]_i_ is modified by dissociation of cells during preparation of cultures. Importantly, in whole DRG we observed higher intracellular Cl concentrations with a mean value of 71.58 mM. This suggests that in this intact preparation with preserved cellular connectivity and architecture, neurons are maintained in a better condition and NKCC1 is able to operate more effectively at supplying neurons with Cl. Similarly, in a study utilizing two-photon fluorescence-lifetime imaging microscopy of the synthetic Cl indicator MQAE in intact DRG, mean [Cl]_i_ was reported to be 77.2 mM (Gilbert et al., 2007).

To confirm that Rosa26::Cl-Sensor mice can also be used to examine [Cl]_i_ in other non-neuronal cell types, mice were crossed with a LysM-Cre driver line to induce expression in cells of the myeloid lineage. We observed robust expression of Cl-Sensor in macrophages that allowed for calibration of the probe and measurement of basal Cl levels. Interestingly, Cl is considered an important anion for the function of macrophages under normal and pathological conditions, and Cl flux has been described in macrophages at rest (Robin et al., 1971; Castranova et al., 1979) and during phagocytosis (Ince et al., 1988). Furthermore, disrupted Cl efflux was demonstrated in macrophages from patients with cystic fibrosis as a result of defective Cl secretion (Doring and Gulbins, 2009). As yet, however, the distribution and functional regulation of [Cl]_i_ in macrophages has not been extensively explored. In our experiments we estimated the mean concentration of Cl in macrophages to be 65.48 mM. This value is relatively high and may reflect enhanced activity of Cl co-transporters or other as yet unknown mechanisms in these cells.

The constructs described here, in line with other fluorescent proteins from the GFP family, exhibit a relatively high sensitivity to pH variations. Earlier we showed that for Cl-Sensor, a shift of pH by 0.1 units results in a change in the ratio for Cl estimation by at most 6% of the whole dynamic range of the probe (Markova et al., 2008). In addition YFP-based molecules are also sensitive to some organic anions (Jayaraman et al., 2000). These points should be taken into account when using these probes and transgenic animals. To circumvent these problems, a combined Cl/pH sensor was recently described (Arosio et al., 2010) which along with its derivatives (Mukhtarov et al., 2013) allows simultaneous ratiometric measurement of these two ions. For these probes, however, transgenic models have not yet been developed.

In conclusion, we have developed transgenic mice that express Cl-Sensor in different cell types, including neurons. The sensitivity and the fact that the probe is genetically encoded should greatly facilitate non-invasive monitoring of [Cl]_i_ and allow for the analysis of Cl transients *in vivo*. Transgenic mice expressing Cl-Sensor under the control of cell-type specific promoters provide a novel tool for the functional characterization of intracellular Cl distribution in defined subsets of neurons in various experimental models.

### Conflict of interest statement

The authors declare that the research was conducted in the absence of any commercial or financial relationships that could be construed as a potential conflict of interest.
