# Supplementary material for: Transgenic mouse lines for non-invasive ratiometric monitoring of intracellular chloride
Source: Front Mol Neurosci. 2013 May 21;6:11. doi: 10.3389/fnmol.2013.00011 (PMC3659292; doi:10.3389/fnmol.2013.00011)
Supplement: Supplementary file 1 [file DataSheet1.PDF]

Figure S1.

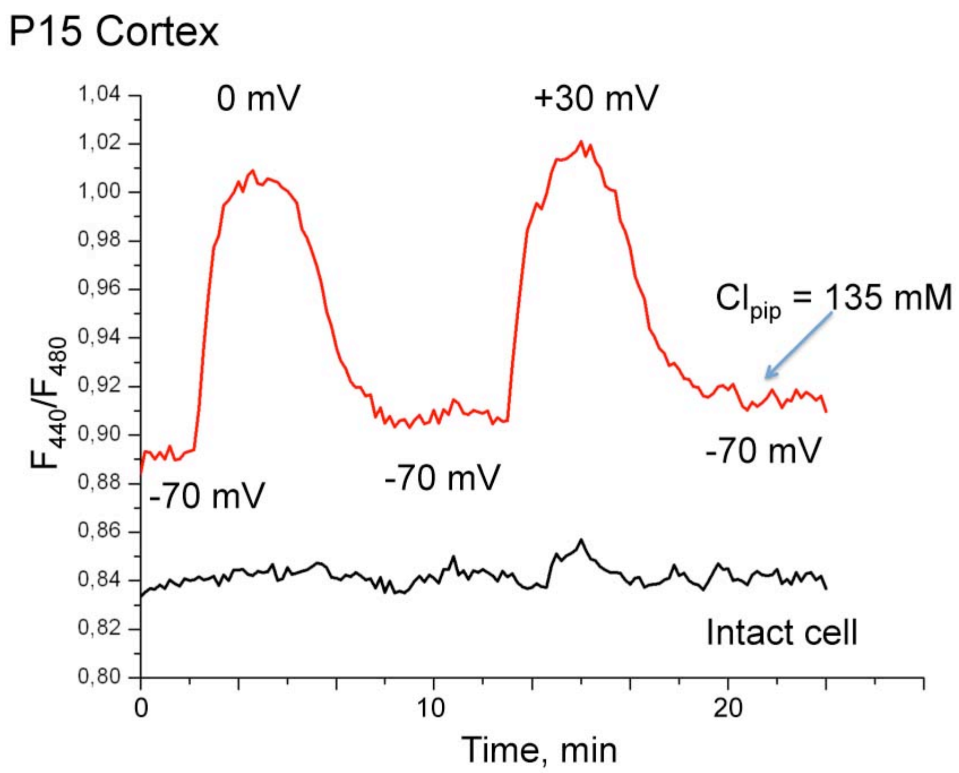

Figure S1. Depolarization to different holding potentials causes similar changes in F_{440}/F_{480}.

Example of changes in R_{Cl} (F_{440}/F_{480}) after membrane depolarization from a holding potential of -70 mV to 0 or +30 mV. *Red trace* - whole-cell recording with pipette containing 135 mM Cl, *Blue trace* - intact cell. Cortex, age P15.
